# Prefrontal dopamine D1 receptor manipulation influences anxiety behavior and induces neuroinflammation within the hippocampus

**DOI:** 10.1186/s40345-020-00212-2

**Published:** 2021-03-08

**Authors:** Dominik K. E. Beyer, Annika Mattukat, Nadja Freund

**Affiliations:** Division of Experimental and Molecular Psychiatry, Department of Psychiatry, Psychotherapy and Preventive Medicine, LWL University Hospital, Ruhr-University, Alexandrinenstr.1, 44791 Bochum, Germany

**Keywords:** Bipolar disorder, Interleukin-6, Anxiety, Lentivirus, Prefrontal cortex, Rat

## Abstract

**Background:**

Prefrontal dopamine D1 receptor (D1R) mediates behavior related to anxiety, reward and memory, and is involved in inflammatory processes, all of which are affected in bipolar disorder. Interleukin-6 (IL-6), a pro-inflammatory cytokine, is increased in patients with bipolar disorder in plasma samples, imaging studies and postmortem tissue and is an indicator for an inflammatory state. We could previously show that lentiviral overexpression of D1R in the medial prefrontal cortex (mPFC) of male adult rats and its termination induces bipolar disorder-like behavior. The purpose of this study was to investigate anxiety and the role of the immune system, specifically IL-6 positive neurons in this animal model. Due to its high density of inflammatory mediator receptors and therewith sensibility to immune activation, the hippocampus was investigated.

**Methods:**

Expression of the gene for D1R in glutamatergic neurons within the mPFC of male, adult rats was manipulated through an inducible lentiviral vector. Animals over-expressing the gene (mania-like state), after termination of the expression (depressive-like) and their respective control groups were investigated. Anxiety behavior was studied in the elevated plus maze and marble burying test. Furthermore, IL-6-positive cells were counted within several subregions of the hippocampus.

**Results:**

D1R manipulation in the mPFC had only mild effects on anxiety behavior in the elevated plus maze. However, subjects after termination buried more marbles compared to D1R over-expressing animals and their respective control animals indicating elevated anxiety behavior. In addition, animals in the depressive-like state showed higher numbers of IL-6 positive cells reflecting an elevated pro-inflammatory state in the hippocampus, in the CA3 and dentate gyrus. Consistently, inflammatory state in the whole hippocampus and anxiety behavior correlated positively, indicating a connection between anxiety and inflammatory state of the hippocampus.

**Conclusions:**

Behavioral and neurobiological findings support the association of manipulation of the D1R in the mPFC on anxiety and inflammation in the hippocampus. In addition, by confirming changes in the inflammatory state, the proposed animal model for bipolar disorder has been further validated.

## Background

The immune system plays an important role in the etiology and pathophysiology of bipolar disorder (BD). BD patients exhibit a persistent and low-grade pro-inflammatory state. This pro-inflammatory state is even more intense during mood episodes, especially manic episodes, and less intense in depressive episodes (Lu et al. 2019; Modabbernia et al. 2013). These findings are supported by phasic differences in the peripheral levels of cytokines in BD patients (Ortiz-Domínguez et al. 2007). Furthermore, even euthymic episodes are associated with increased peripheral pro-inflammatory cytokines (Brietzke et al. 2009a; Brietzke et al. 2009b). Overall, abnormalities of the immune system have been linked to symptom severity (Goldstein et al. 2009), state of mood episodes (Brietzke et al. 2009b; Ortiz-Domínguez et al. 2007) and treatment effect (Benedetti et al. 2017; Goldstein et al. 2009). These findings are supported by the evidence, that adjunctive treatment with some anti-inflammatory agents is able to alleviate manic and improve depressive symptoms (Goldsmith et al. 2016; Köhler et al. 2014). Interestingly, pro-inflammatory cytokines can also be attenuated by lithium (Himmerich et al. 2013; Patel and Frey 2015).

One of the most reported pro-inflammatory cytokines is interleukin-6 (IL-6). IL-6 plays a key role in the acute phase response and mediates the transition from acute to chronic inflammation (Gabay 2006; Marin et al. 2001).

Scores of the Young Mania Rating Scale and the Hamilton Depression scale correlated with IL-6 (Brietzke et al. 2009b). IL-6 is increased in acute manic and euthymic episodes compared to controls, whereas IL-6 levels were not significantly different between bipolar depressed patients and healthy controls (Goldsmith et al. 2016). Different serum levels of pro-inflammatory cytokines, such as IL-6, might be a potential biomarker to distinguish between major depressive disorder (MDD) and BD, whereby IL-6 was elevated in BD but not in MDD patients compared to healthy controls (Lu et al. 2019). In contrast to the findings from Lu et al. (2019), IL-6 levels have been shown to be increased in unipolar depressed patients as well (Goldsmith et al. 2016). It is even discussed, that IL-6 antagonists could be potential therapeutic agents for BD patients (Brietzke et al. 2011). Overall, a lot of evidence indicates, that BD and aberrant immune-inflammatory pathways are highly related to each other. The exact molecular mechanisms and connections between BD and the immune system, however, are not fully understood.

Interestingly, BD is linked to dysregulated dopamine (DA) signaling. In addition, the relationship of DA, DA receptors and inflammation is widely known (Xia et al. 2019). In summary, DA and its receptors are able to regulate the immune system in an activating and inhibiting manner (Sarkar et al. 2010). One of those immunoregulatory DA receptors is the dopamine D1 receptor (D1R). DA and D1R signaling via the second messenger cyclic adenosine monophosphate (cAMP) prevent nucleotide-binding oligomerization domain-like receptor pyrin domain-containing 3 (NLRP3) inflammasome-dependent inflammation, including lipopolysaccharide (LPS)-induced systemic inflammation and neurotoxin-induced neuroinflammation in vivo (Yan et al. 2015).

The relationship between DA levels, its receptors and the immune system is bidirectional. Indeed, immune system activation and the resulting release of inflammatory cytokines can negatively affect reward behavior, motivation, anxiety and DA, which can be identified as depression-like behavior in animals (Remus and Dantzer 2016) and depressive symptoms in patients (Capuron et al. 2012; Eisenberger et al. 2010).

A well-established method to study consequences of innate immune system activation is the administration of LPS. LPS treatment promotes an activation of immune-inflammatory pathways, accompanied by the release of pro-inflammatory cytokines, such as IL-6 in the brain and periphery (Qin et al. 2007; Sulakhiya et al. 2016). Behavioral consequences are higher anxiety and depression-like behavior (Davis et al. 2017; Skurlova et al. 2011; Sulakhiya et al. 2016). In addition, a chronic pro-inflammatory state can lead to behavioral abnormalities, such as aberrant anxiety behavior (Michopoulos et al. 2017). Induction of depression-like behavior via learned helplessness paradigm furthermore resulted in an increased IL-6 expression within the hippocampus (Onufriev et al. 2017). Additionally, it is discussed, that Ketamine’s antidepressant effect might be mediated by its ability to reduce cytokines in the hippocampus and therefore acting anti-inflammatory (Wang et al. 2015) connecting increased inflammatory states in the hippocampus with depression-like behavior.

Here, we used an animal model, which was capable of inducing mania- and depression-like behavior within the same rat in an alternating manner. Viral D1R over-expression in the medial prefrontal cortex (mPFC) of rats resulted in mania-like behavior (Freund et al. 2016; Sonntag et al. 2014). Termination of viral D1R over-expression alone was sufficient to induce depression-like behavior (Freund et al. 2016). Based on the importance of hippocampal inflammatory state as outlined above we investigated an aberrant activation of the immune system, via analyzing the number of IL-6-positive cells in the hippocampus.

## Methods

### Animals

A total of n = 24 adult, male Sprague Dawley rats were obtained from Charles River Laboratories (Sulzfeld, Germany). Rats were pair-housed (2 animals per cage) in a standard cage with food and water available ad libitum in constant temperature and humidity conditions (22 ± 2 °C and 55 ± 25%) on a 12-h light/12-h dark cycle (light period 23:00–11:00). The animals were allowed to acclimate to the animal facility for 1 week before surgery. Only males were used in order to avoid an influence of the estrous cycle on dopaminergic processes within the PFC, nucleus accumbens and striatum of female rats (Kazandjian et al. 1988). All experiments were carried out in agreement with the principles regarding the care and use of animals adopted by the German Animal Welfare Law for the prevention of cruelty to animals after approval by the LANUV (Landesamt für Natur, Umwelt und Verbraucherschutz Northrhine-Westfalia).

### Lentiviral vector

#### Virus production

The same lentiviral construct was used as previously described (Freund et al. 2016). In short, a Tetracycline-On inducible lentiviral vector system (Tet.On) was used, coding for the rat D1R protein (*DRD1* gene) or for control condition for the red fluorescent protein dsRed. The system, was driven by a CamKIIα promoter to constitutively drive rtTA3 expression and dsRed or D1R expression was regulated by the tetracycline derivative doxycycline (DOX). Virus production was performed by the Viral Core Facility, Charité Universitätsmedizin Berlin based on the protocol of Stewart et al. (2003) with the use of plasmids 8454 and 8455 by Addgene.

#### Surgery

Rats were anesthetized with ketamine/xylazine/azaperone mixture (100/16/8 mg/kg) and 1 µl of virus (2 × 10^7^ transducing units per µl) were bilaterally injected into the mPFC at stereotaxic coordinates (AP: + 2.7, ML: ± 0.4, DV: − 2.8) of rat brain atlas (Paxinos et al. 1980).

#### Doxycycline treatment

**Fig. 1:**
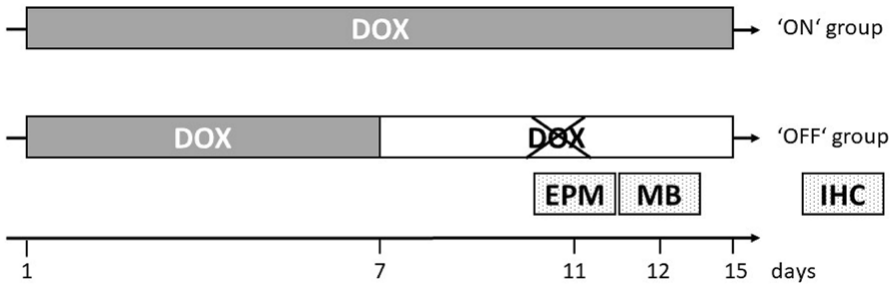
Experimental design. Glutamatergic neurons in the mPFC of rats were transduced with D1R or dsRed. After recovery from virus injection rats were treated for 7 continuous days with DOX to induce virus over-expression. In one half of the subjects, virus over-expression was maintained and is referred to as ‘ON’. The ‘OFF’ group was not further provided with DOX after initial 7 days of virus over-expression. Behavioral experiments (EPM: Elevated Plus Maze and MB: Marble burying) were performed on day 11 and 12, respectively. Subjects were sacrificed via perfusion on day 15 and immunohistochemistry (IHC) was performed afterwards

The virus system can be controlled through administration of DOX hyclate (Sigma-Aldrich). Virus expression was activated by 0.5 g/l DOX in the drinking water. As previously shown, full viral expression occurred within seven days after DOX administration (‘ON’ state) and viral over-expression was reduced three days after DOX removal (‘OFF’ state) (Freund et al. 2016). ‘ON’ group received in total 15 days of DOX through drinking water, whereas the ‘OFF’ group was given DOX for 7 days followed by 8 days of regular drinking water without DOX (Fig. 1), resulting in a total of 4 groups: ‘D1R ON’, ‘D1R OFF’, ‘dsRed ON’ and ‘dsRed OFF’. Each group consisted of 6 rats.

After virus injection, rats received DOX on 7 continuous days to induce virus over-expression. Virus over-expression was maintained in one half of the subjects till their sacrifice. This group is referred to as ‘ON’ group and ‘D1R ON’ demonstrated mania-like behavior in previous experiments (Freund et al. 2016). The ‘OFF’ group received 7 days of DOX following DOX removal till immunohistochemistry. ‘D1R OFF’ subjects demonstrated depression-like behavior (Freund et al. 2016). Anxiety-related behavior was investigated in all 4 groups on day 11 via elevated plus maze and through marble burying on day 12. Subjects were sacrificed on day 15.

### Behavioral assessments

#### Elevated plus maze (EPM)

EPM was performed on day 11. The EPM is a paradigm to test anxiety in animals, consists of two open and closed arms (each 15 × 42.5 cm) and was raised 72.5 cm from the floor. Tests were performed during the dark phase of the light–dark cycle under dim red light conditions. Each subject, experimentally naïve, was placed in the central platform of the maze, facing a closed arm, and allowed to freely explore the maze for 5 min. Time spent in the open arms, open arm entries and time until first open arm entry were recorded to assess anxiety. Crossings and general arm entries were analyzed to evaluate general motor activity. An investigator blind in terms of experimental conditions performed scoring.

#### Marble burying test (MB)

Marble burying test was used to investigate anxiety (Jimenez-Gomez et al. 2011; Kedia and Chattarji 2014). The test was conducted on day 12 during the dark phase of the light–dark cycle under dim red light conditions in unfamiliar standard cages, containing a 5 cm layer of bedding. Twenty marbles were evenly placed in rows on the surface of the bedding. Rats were placed in the cage for 15 min. The number of totally buried marbles were counted for each rat.

#### Immunohistochemistry (IHC), cell counting and analysis of IL-6 staining

For IHC rats were deeply anesthetized with a ketamine/xylazine mixture (100/10 mg/kg) and intracardially perfused with 4% paraformaldehyde as previously described (Gage et al. 2012). The brains were cryoprotected and cut into 40 µm coronal sections. After blocking in 10% normal goat serum, sections were exposed overnight at 4 °C to 1:300 rabbit anti-IL-6 (Proteintech), washed in 1x PBS, and incubated for 60 min at room temperature with anti-rabbit Alexa 488-coupled IgG (Sigma-Aldrich). Sections were washed, incubated with 20 µg/ml 4′,6-diamidino-2-phenylindole (DAPI), washed, and mounted on slides. Images of the regions of interest within the hippocampus (bregma − 2.76 mm) (Fig. 3a) were generated with a fluorescence microscope (Olympus BX51) and the software CellD under consistent exposure time. Regions of interest were hippocampal subregions CA1, CA2, CA3, CA4, DG and were adapted to an image size of 300 × 900 pixels via GIMP (version 2.10.14). IL-6-positive cells were identified as a completely filled cell with fluorescence signal. Cells were counted manually by an investigator blinded in terms of experimental conditions with ImageJ (Schindelin et al. 2012) (Table 1).

**Table 1:** Number of IL-6-positive cells in subregions of the hippocampus following D1R manipulation within the mPFC

	D1R ‘ON’	dsRed ‘ON’	D1R ‘OFF’	dsRed ‘OFF’
CA1	52.7 ± 11.6	31.8 ± 12.5	53 ± 15.6	42.3 ± 6.4
CA2	37.9 ± 5.9	38 ± 8.8	48 ± 12.2	46.5 ± 3.3
CA3	18 ± 6.5	5.9 ± 4.5	36 ± 9.9	11.3 ± 2
CA4	14.2 ± 5.3	27.2 ± 10.5	24.5 ± 9.8	9.7 ± 4.7
DG	11.3 ± 4.8	28.5 ± 9.6	33 ± 14.2	18 ± 8.7

Means and standard errors are listed

#### Statistics

Statistics were performed with SPSS statistical software 26 (version 26.0.0.0). Data are depicted as means and SEMs. Behavioral data was analyzed with a two-way analysis of variance (ANOVA) with virus group (D1R and dsRed) and virus state (‘ON’ and ‘OFF’) as factors. Based on our previous behavioral results (Freund et al. 2016; Sonntag et al. 2014), we hypothesized that D1R ‘ON’ subjects demonstrate mania-like behavior whereas D1R ‘OFF’ animals show depression-like behavior. Due to this a priori prediction one-sided t-test with Bonferroni correction was used as post-hoc test for behavioral results. Immunohistochemistry including cell counting of IL-6 and DAPI-positive cells were analyzed for each subregion separately via two-way ANOVA with virus group and virus state as factors. Post-hoc comparison was performed in all cases via Bonferroni corrected independent t-test. Correlation between IL-6 immunoreactivity and anxiety behavior was assessed with Pearson correlation coefficients. Experimenters were blind to treatment of animals during experimental and data collection processes. In all cases, p < 0.05 was considered as statistically significant.

## Results

### Elevated plus maze

**Fig. 2:**
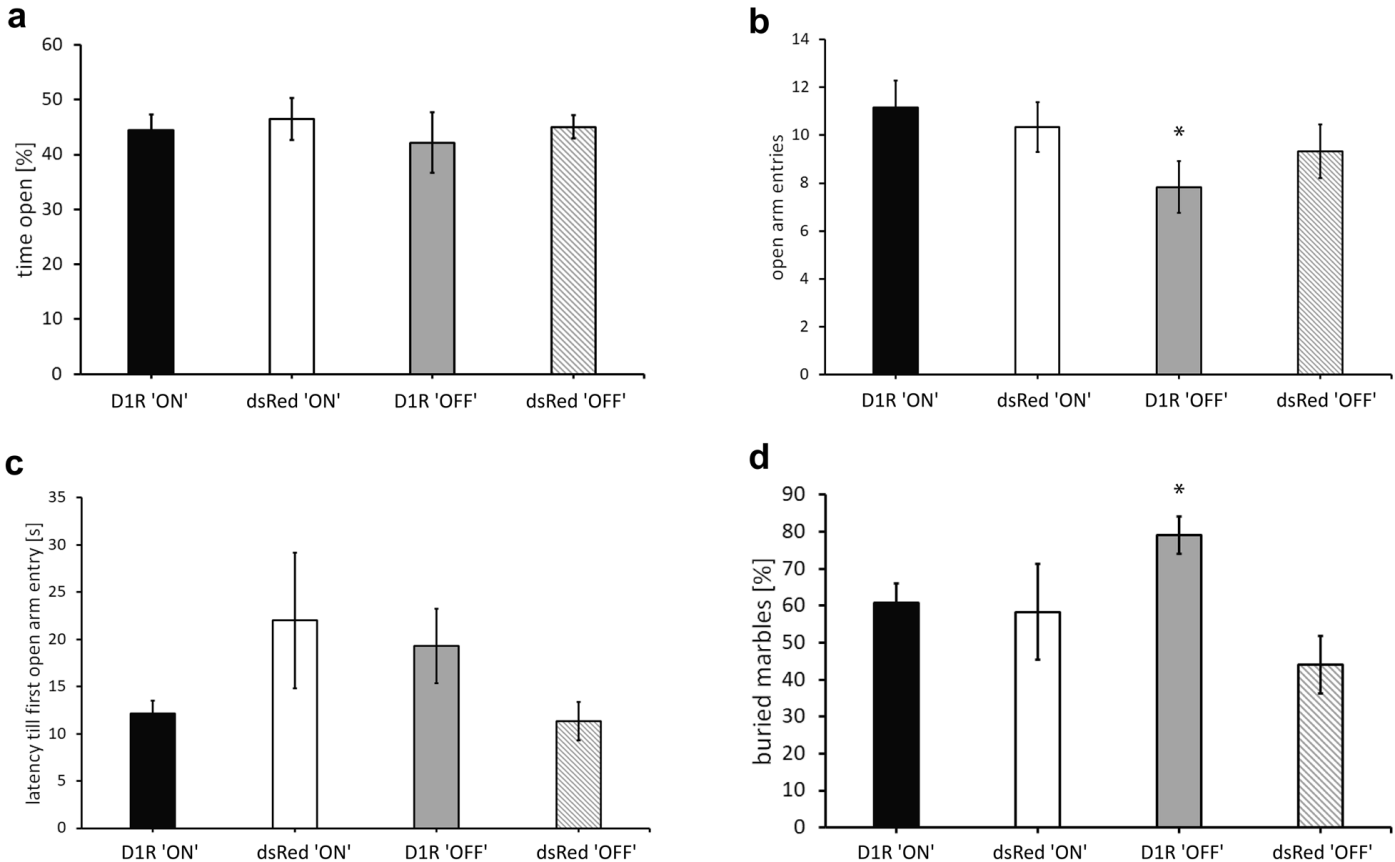
Anxiety behavior and compulsive activity accessed in the Elevated Plus Maze and Marble Burying paradigm indicated through time in open arm, open arm entries, latency till first open arm entry and amount of buried marbles. Virus over-expression (‘ON’) is terminated (‘OFF’) following doxycycline removal. **a** Anxiety as measured time spent in the open arm [%]. **b** Anxiety behavior was also determined as number of open arm entries. **c** Risky behavior as measured as latency till first open arm entry. **d** D1R ‘OFF’ animals buried significantly more marbles than their control group (dsRed ‘OFF’) and D1R ‘ON’ subjects. Means ± SEM are presented for n = 6 subject for each group. *Represent p < 0.05 Bonferroni correction indicating significant differences between relevant groups

Time spent in the open arm did not reveal any differences between groups. Neither a main effect of the transduced virus (F(1,20) = 0.4, p = 0.5), nor virus state main effect F(1,20) = 0.25, p = 0.6), nor an interaction of virus group × virus state were observable (F(1,20) = 0.014, p = 0.9) (Fig. 2a). Open arm entries showed no main effect of virus (F(1,20) = 0.67, p = 0.13) but virus state (F(1,20) = 28.167, p = 0.028) and no significant virus state × virus group interaction (F(1,20) = 8.167, p = 0.21) (Fig. 2b). Post-hoc comparison revealed lower anxiety behavior, indicated through more open arm entries in the ‘ON’ groups (Bonferroni correction, p < 0.05) and a trend of fewer open arm entries in the ‘D1R OFF’ compared to ‘D1R ON’ group. Time until first entry into open arm was not affected by virus group (F(1,20) = 0.5, p = 0.8) nor virus state (‘F(1,20) = 0.17, p = 0.69) but a significant virus group × virus state interaction was found (F(1,20) = 4,378, p = 0.049). This result, however, was not able to withstand post-hoc comparison via Bonferroni correction between all groups (Bonferroni correction, p > 0.05) (Fig. 2c). General motor activity measured as crossings in the EPM was not effected by virus group, (F(1,20) = 0.58, p = 0.46), virus state F(1,20) = 0.007, p = 0.9) or virus group × virus state interaction (F(1,20) = 0.07, p = 0.8). In addition, general arm entries were not influenced by virus group (F(1,20) = 0.08, p = 0.79), virus state F(1,20) = 1.3, p = 0.27) or virus group × virus state interaction (F(1,20) = 1.1, p = 0.3). Taken together, D1R over-expression in the mPFC had only a mild influence on anxiety in the elevated plus maze. Termination of D1R over-expression resulted in a higher latency until first open arm entry and reduced number of open arm entries. General locomotor activity was not affected.

### Marble burying

The number of buried marbles was affected by virus group (F(1,20) = 5.025, p = 0.036), but not virus state (F(1,20) = 0.6, p = 0.8) and a trend for an interaction of virus group × virus state was found (F(1,20) = 3.774, p = 0.066). Post-hoc comparison revealed, that D1R animals buried significantly more marbles than dsRed animals (Bonferroni correction, p < 0.05). D1R ‘OFF’ animals furthermore buried significantly more marbles than subjects of their control group, dsRed ‘OFF’ (Bonferroni correction, p < 0.05). Marble burying revealed that D1R manipulation increases anxiety behavior in general, however elevated anxiety behavior was mainly demonstrated after the termination of the over-expression of D1R (Fig. 2d).

### IL-6 IHC in the hippocampus

**Fig. 3:**
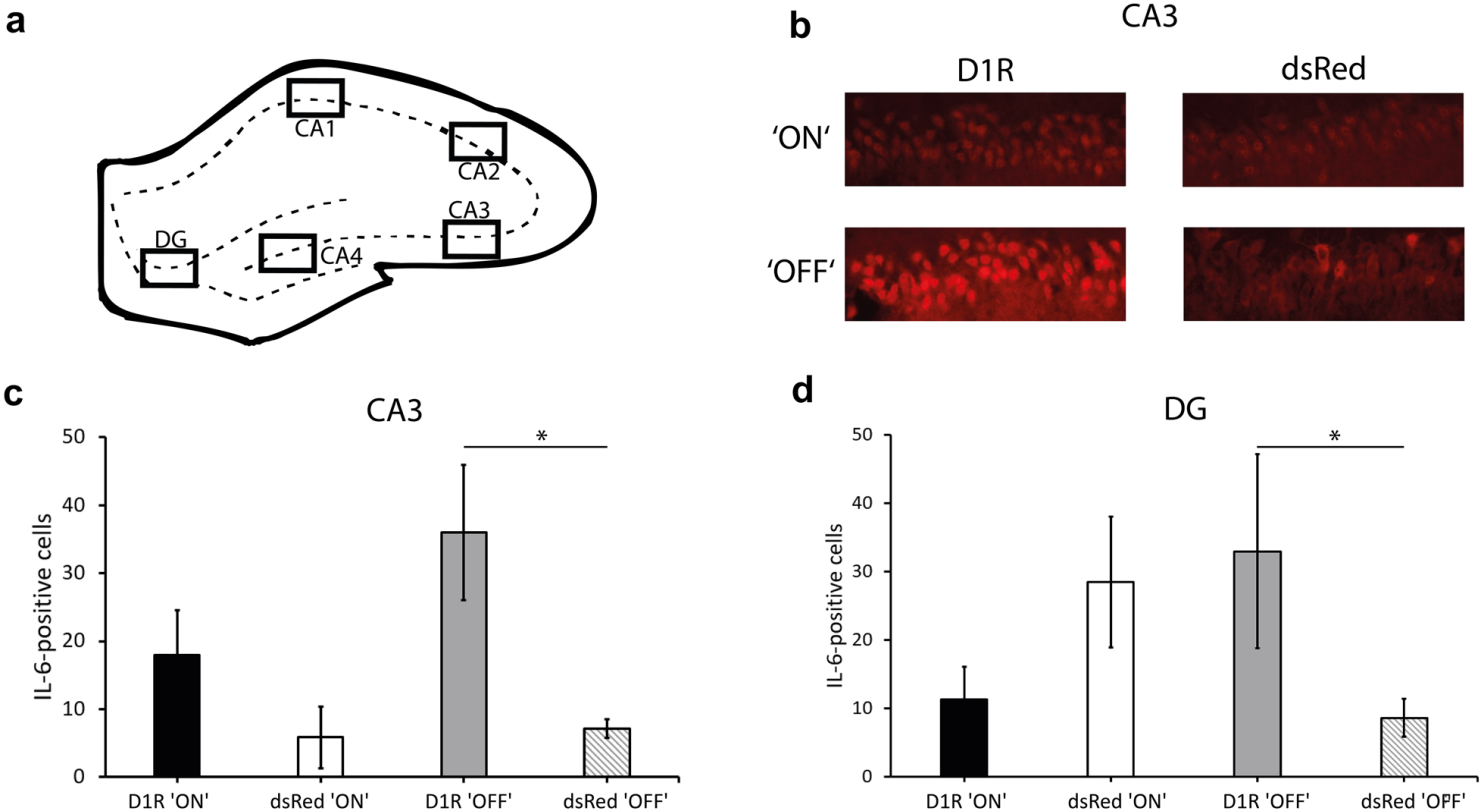
IL-6-positive cells. Virus over-expression (‘ON’) is terminated (‘OFF’) following doxycycline removal. **a** Schematic representation of the hippocampus including the regions of interest (hippocampal CA1, CA2, CA3, CA4 and DG). **b** Representation of IL-6 staining in the CA3 region of the hippocampus. **c** Amount of IL-6-positive cells in the CA3 is significantly increased in D1R ‘OFF’ subjects compared to controls. **d** No significant differences in the DG were observable between groups. Means ± SEM are presented for n = 6 subject for each group. *Represent p < 0.05 Bonferroni correction indicating significant differences between relevant groups

Virus group did not affect the amount of IL-6-positive cells in the CA1 (F(1,20) = 2.16, p = 0.16), CA2 (F(1,20) = 0.3, p = 0.87), CA4 F(1,20) = 0.003, p = 0.9) and DG (virus group main effect: F(1,20) = 0.15, p = 0.7). However, virus group had a significant effect on IL-6-positive cells in the CA3 region of the hippocampus F(1,20) = 9.399, p = 0.006) (Fig. 3b, c). The number of IL-6-positive cells were significantly elevated in D1R ‘OFF’ compared to subjects transduced with dsRed (Bonferroni correction, p < 0.05). No subregion showed a main effect of virus state (CA1: F(1,20) = 0.02, p = 0.9, CA2: F(1,20) = 0.62, p = 0.44, CA3: F(1,20) = 2.1, p = 0.16, CA4: F(1,20) = 0.07, p = 0.79, DG: F(1,20) = 0.01, p = 0.9). A significant virus group × virus state interaction revealed an influence of virus over-expression and its termination in the mPFC on IL-6-positive cells only in the DG (F(1,20) = 5.12, p = 0.035) but not the other subregions (CA1: F(1,20) = 0.1, p = 0.9, CA2: F(1,20) = 0.04, p = 0.8), CA3: F(1,20) = 1.6, p = 0.23 and CA4: F(1,20) = 2.3, p = 0.15) (Fig. 3d). However, differences between groups were not big enough to withstand Bonferroni correction (Bonferroni correction, p > 0.05). In addition, no interaction of virus group × virus state were observable in CA1 (F(1,20) = 0.1, p = 0.9), CA2 (F(1,20) = 0.04, p = 0.8), CA3 (F(1,20) = 1.6, p = 0.23) and CA4 (F(1,20) = 2.3, p = 0.15).

To determine if inflammation changed due to differences of total cell numbers, DAPI-positive cells were analyzed. The amount of DAPI-positive cells did not differ between virus groups (CA1: F(1,20) = 0.3 p = 0.58, CA2: F(1,20) = 0.09, p = 0.77, CA3: F(1,20) = 0.01, p = 0.92, CA4: F(1,20) = 2.3, p = 0.15 and DG: F(1,20) = 0.0, p = 0.99) or virus states (CA1: F(1,20) = 2.3, p = 0.14, CA2: F(1,20) = 1.5, p = 0.24, CA3: F(1,20) = 0.06, p = 0.8, CA4: F(1,20) = 0.004, p = 0.95 and DG: F(1,20) = 1, p = 0.33). In addition, there was no significant interaction of virus group × virus state (CA1: F(1,20) = 0.7, p = 0.4, CA2: F(1,20) = 0.003, p = 0.96, CA3: F(1,20) = 0.07, p = 0.79, CA4: F(1,20) = 0.2, p = 0.68 and DG: F(1,20) = 1.1, p = 0.3).

### Correlation between IL-6 immunoreactivity and anxiety behavior

**Fig. 4:**
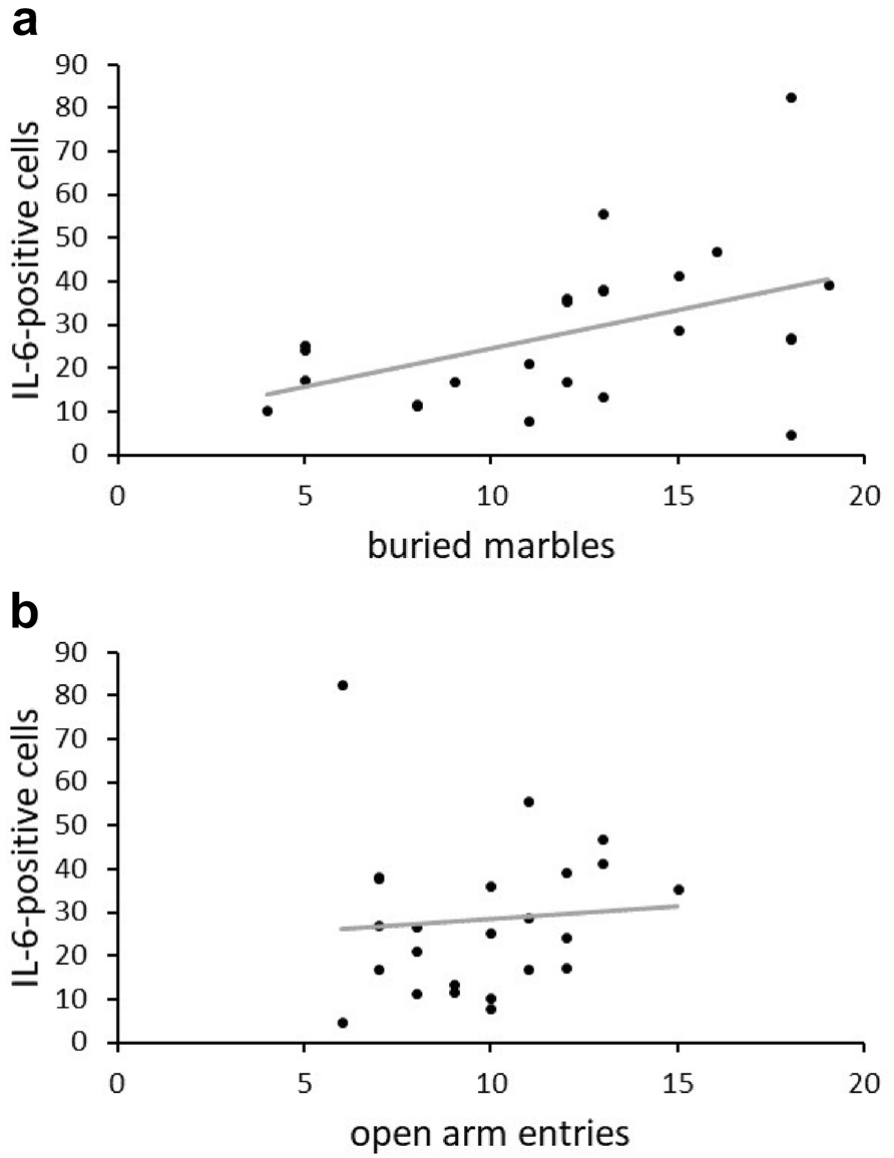
**a** The amount of IL-6-positive cells in the whole hippocampus correlate significantly positive with the amount of buried marbles representing anxiety behavior. **b** No significant correlation of IL-6-positive cells in the whole hippocampus with the amount of open arm entries in the EPM. Each dot represents one subject (n = 24)

To further analyze if IL-6 as a marker for a pro-inflammatory state and anxiety behavior are connected, a correlation between IL-6-positive cells and buried marbles was performed (Fig. 4a). Indeed, an increased number of IL-6-positive cells in the whole hippocampus was connected to an elevated anxiety behavior indicated through more buried marbles (r = 0.461, p = 0.023). Interestingly, number of IL-6-positive cells in the whole hippocampus were not associated with increased anxiety behavior measured as open arm entries in the EPM (r = 0.08, p = 0.7) (Fig. 4b).

## Discussion

Our results demonstrate an increase of inflammatory activation in the hippocampus, specifically in the CA3 and DG area, following D1R over-expression and its termination in glutamatergic neurons of the mPFC. The manipulation had no effect on the total number of cells indicating that elevated IL-6 represents higher inflammatory processes in the same neurons and our results are not based on a change of neuronal cell quantity.

The PFC is innervated by dopaminergic projections and modulates multiple regions in the brain that are associated with depression, including the hippocampus. Our findings support an effect of D1R manipulation on the immune system within the hippocampus. Hippocampal neurons are key contributors to anxiety and depression. Depressive-like behavior can e.g. be prevented by stimulation of BDNF expression (Wang et al. 2016) in the hippocampus or even by inhibition of hippocampal inflammation (Zhou et al. 2018; Zhou et al. 2017). BD patients demonstrate decreased PFC and increased amygdala activity on emotional cues (Lawrence et al. 2004), which in turn is associated with increased IL-6 release (Felger 2018; Muscatell et al. 2015). The amygdala innervates the hippocampus and might thereby increase the inflammatory state in this brain region, which is associated with affective disorders. This could be a possible mechanism for our findings of the connection of D1R manipulation in the mPFC and inflammatory state in the hippocampus.

Aberrant neuroinflammation creates severe consequences. For instance, IL-6 overproduction leads to neurodegeneration (Campbell et al. 1993), and blocking of IL-6 signaling alleviate harmful IL-6 effects in the brain (Campbell et al. 2014). Here we did not see any differences in total cell number, even though subjects after the termination of D1R over-expression (D1R ‘OFF’) showed an elevated number of IL-positive cells. This result indicates that aberrant immune activation was probably too short in our experimental setup to induce neurodegeneration.

There is clear evidence suggesting that activation of the immune system contributes to the etiology and manifestation of anxiety and depression (Miller et al. 2013; Vogelzangs et al. 2013). Interestingly, no effect of D1R manipulation in the mPFC of rats on anxiety measured by time spent in open arms in the EPM paradigm was present in this study. However, latency until first entry and number of entries into open arms, seemed to be increased and therewith reflect anti-anxiety behavior (Walf and Frye 2007) in the D1R over-expressing group. In line with our previous findings (Freund et al. 2016; Sonntag et al. 2014), this trend for anti-anxiety behavior in D1R ‘ON’ subjects can even be interpreted as mania associated behavior. In addition, D1R manipulation was able to influence anxiety behavior in the MB paradigm. Termination of D1R over-expression (D1R ‘OFF’) resulted in higher anxiety behavior in subjects compared to control (dsRed ‘OFF’) and D1R over-expression animals (D1R ‘ON’). These results are in line with previous experiments with the same animal model, whereby D1R ‘OFF’ subjects revealed a depression-like phenotype (Freund et al. 2016). Anxiety and depression-like behavior were often accompanied by a pro-inflammatory state in the hippocampus (Liu et al. 2019; Sulakhiya et al. 2016; Xu et al. 2016). Pharmacological treatment with agents reducing inflammation were able to alleviate both behavioral symptoms: depression-like behavior and anxiety (Liu et al. 2019; Sulakhiya et al. 2016; Zager et al. 2018). Inhibiting IL-6 expression in the hippocampus plays a key role for the implementation of these beneficial effects (Skurlova et al. 2011; Sulakhiya et al. 2016). Affected inflammation and anxiety and depression-like behavior in rodents, has been shown by several manipulations such as sleep deprivation (Wadhwa et al. 2018; Yin et al. 2017), chronic mild stress (Wang et al. 2018) and models for chronic inflammatory disease, like diabetes or metabolic syndrome (Zhou et al. 2018). All those findings share, that increased inflammation in the hippocampus induces anxiety and depression-like behavior in rodents, and those behavioral symptoms could be attenuated through anti-inflammatory agents which reduced among other pro-inflammatory cytokines IL-6. According to the increased anxiety in D1R ‘OFF’ subjects is the trend, that these animals spent more time in the closed arm until they entered one of the open arms for the first time compared to D1R ‘ON’ and dsRed ‘OFF’ animals. This may indicate a decreased risk-taking and novelty-seeking behavior in D1R ‘OFF’ animals. Contrariwise, it means that D1R ‘ON’ subjects displayed an increased risk-taking behavior in comparison to the D1R ‘OFF’ group. This is in line with findings of decreased anxiety and increased risk-taking behavior following D1R virus over-expression (Sonntag et al. 2014) or optogenetic stimulation of mPFC D1R neurons (Hare et al. 2019). Zager and colleagues investigated Modafinil’s role, a psychostimulant for treating narcolepsy, excessive daytime sleepiness, sleep apnea and shift work, on LPS-induced neuroinflammation. Modafinil was able to prevent LPS-induced anxiety and depression-like behavior and inflammation. Interestingly, D1R signaling was necessary for Modafinil’s mechanism of action on locomotion and anxiety (Zager et al. 2018) supporting a further direct link of the D1R and the immune system.

The link between D1R signaling and the immune system combined with our results are in line with previous in vitro findings, that the absence of D1R induced neuroinflammation (Yan et al. 2015). One potential mechanism of action of this animal model for BD is a decreased endogenous expression of D1R following its viral over-expression. Termination of this over-expression leads to behavior which can be described as depression-like (Freund et al. 2016). A reduction of D1R could therefore be an explanation for increased inflammation within D1R ‘OFF’ subjects. There is a strong association of both chronic pro-inflammatory states in patients of BD (Muneer 2016; Stertz et al. 2013), MDD (Berk et al. 2013; Miller et al. 2009), anxiety (Miller et al. 2013; Pace and Heim 2011) and dopaminergic dysfunction (Berk et al. 2007). These findings are supported by the evidence, that adjunctive treatment with some anti-inflammatory agents is able to alleviate manic and improve depressive symptoms (Goldsmith et al. 2016; Köhler et al. 2014; Miller et al. 2017; Rosenblat 2019), whereas anti-inflammatory agents primarily improved motivation and anxiety in animal studies (Raison et al. 2013). Interestingly, many antidepressant drugs cause anti-inflammatory effects (Hannestad et al. 2011). In addition, our findings match with first results on the antipsychotic drug Lumateperone, which is tested for a wide range of neuropsychiatric disorders. Lumateperone has serotonergic, glutamatergic and dopaminergic pharmacological effects, and is able to bind to D1R (Kumar et al. 2018). First clinical trials showed an improvement of depressive symptoms in bipolar depression patients (unpublished data). Manipulation of D1R and its effect on mood could be a shared mechanism between Lumateperone and the here described animal model for BD.

In addition, we found a correlation of IL-6-positive cells in the whole hippocampus and anxiety behavior. Indeed, antidepressants attenuate depression-like behavior and reduce pro-inflammatory cytokines within the hippocampus (Liu et al. 2017; Wang et al. 2015). Our data in combination with those findings further support this manipulation as a potential animal model for BD. This is supported by the fact that depressive BD patients show anxiety related comorbidities (Vázquez et al. 2014) and increased anxiety behavior is one characteristic feature of animal models of depression (Beyer and Freund 2017; Wang et al. 2017).

One limitation of this study is the usage of DOX. It should be taken into account that inflammatory activation indicated through IL-6-positive cells should be considered higher in ‘ON’ subjects, especially in the D1R ‘ON’ group. This estimation is based on the fact, that these subjects were treated with DOX, an antibiotic, that decreases inflammatory processes and thereby IL-6 production and signaling (Di Caprio et al. 2015). One additional limitation is the investigation of IL-6 as the only pro-inflammatory cytokine and not the investigation of different anti-inflammatory cytokines. Further investigations of several pro- and anti-inflammatory cytokines are necessary. Nonetheless, our results are a first hint for the connection of D1R manipulation in the mPFC and neuroinflammation in the hippocampus.

To sum up, D1R stimulation in the mPFC results in mania-like behavior (Freund et al. 2016; Hare et al. 2019; Sonntag et al. 2014) and termination of previous viral over-expression in depression-like behavior. Here, we demonstrated a link between D1R manipulation, increased inflammation in the hippocampus and anxiety.

## Conclusions

This study shows, that D1R over-expression within the rats’ mPFC neither significantly affects anxiety behavior nor inflammation in the brain. Interestingly, termination of viral D1R over-expression alone was sufficient enough to induce increased anxiety and inflammation in the hippocampus thereby providing a connection between D1R manipulation, increase of inflammational activation in the brain and anxiety. We hypothesize, that D1R over-expression induces a pro-inflammatory state, which is kept at low level due to administration of DOX. Termination of DOX enhances inflammatory processes and induce behavioral abnormalities resembling depression-like behavior. Therefore, this chronic inflammation may lead to a BD-like phenotype in combination with DOX’s properties of inhibiting inflammation. The behavioral findings in combination with molecular results strengthens the described animal model as a very promising animal model for BD (Beyer and Freund 2017).

## Data Availability

Data is made available upon request.

## Abbreviations

BD: Bipolar disorder
BDNF: Brain-derived neurotrophic factor
CaMKII: Calmodulin kinase II
CREB: Cyclic AMP-responsive element binding protein
DA: Dopamine
DAPI: 4′,6-Diamidino-2-phenylindole
DG: Dentate gyrus
DOX: Doxycycline
D1R: Dopamine D1 receptor
EPM: Elevated plus maze
IHC: Immunohistochemistry
IL-6: Interleukin-6
LPS: Lipopolysaccharide
MANOVA: Multivariate analysis of variance
MDD: Major depression disorder
mPFC: Medial PFC
NLRP3: Nucleotide-binding oligomerization domain-like receptor pyrin domain-containing 3
Tet.On: Tetracycline-On inducible lentiviral vector system
